# ^89^Zr-Radiolabelling of p-NCS-Bz-DFO-Anti-HER2 Affibody Immunoconjugate: Characterization and Assessment of In Vitro Potential in HER2-Positive Breast Cancer Imaging

**DOI:** 10.3390/pharmaceutics17060739

**Published:** 2025-06-04

**Authors:** Maria-Roxana Tudoroiu-Cornoiu, Radu Marian Șerban, Diana Cocioabă, Dragoș Andrei Niculae, Doina Drăgănescu, Radu Leonte, Alina Catrinel Ion, Dana Niculae

**Affiliations:** 1 Radiopharmaceutical Research Centre (CCR), Horia Hulubei National Institute for R&D in Physics and Nuclear Engineering (IFIN-HH), 30 Reactorului Street, 077125 Măgurele, Romania; roxana.cornoiu@nipne.ro (M.-R.T.-C.); radu.serban@nipne.ro (R.M.Ș.); radu.leonte@nipne.ro (R.L.); dana.niculae@nipne.ro (D.N.); 2 Faculty of Chemical Engineering and Biotechnologies, Doctoral School of Applied Chemistry and Materials Science, National University of Science and Technology Politehnica Bucharest, 1-7 Gheorghe Polizu Street, 011061 Bucharest, Romania; 3 Faculty of Pharmacy, Carol Davila University of Medicine and Pharmacy, 6 Traian Vuia Street, 020956 Bucharest, Romania

**Keywords:** zirconium-89, pharmacokinetics, radiopharmaceuticals, HER2-positive, affibody, breast cancer

## Abstract

**Background:** The ^89^Zr radioisotope is increasingly vital in positron emission tomography (PET), especially immuno-PET, due to its long half-life of 78.4 h, allowing extended tracking of biological processes. This makes it particularly suitable for researching medicines with slow pharmacokinetics and enhances the precision of molecular imaging, especially in oncology. Despite zirconium’s potential for skeletal accumulation, effective chelation with agents like deferoxamine (DFO) enables high-resolution imaging of antigen-specific tumours, such as HER2-positive breast cancer, offering insights into tumour biology and treatment response. **Methods**: ^89^Zr was produced at the ACSI TR-19 cyclotron via ^89^Y(p,n)^89^Zr reaction. Natural yttrium foils (250 μm) were irradiated with 12.9 MeV protons on target, with 100 μA·h. An HER2-targeting affibody was synthesized and conjugated with p-NCS-Bz-DFO (1:4 mass ratio) at 37 °C for 60 min (pH 9.2 ± 0.2), then purified on a PD-10 column. Radiolabelling was performed with [^89^Zr]Zr-oxalate at pH ranging from 7.0 to 9.0, with concentrations from 110 to 460 MBq/mL. **Results**: Final activity reached 2.95 ± 0.31 GBq/batch (EOB corrected), with ≥ 99.9% radionuclide and ≥95% radiochemical purities. The anti-HER2 affibody was successfully radiolabelled with ^89^Zr, resulting in a radiochemical purity of over 85% with molar activity of 26.5 ± 4.4 and 11.45 MBq/nmol at pH 7.0–7.5. In vitro tests on BT-474 and MCF-7 cell lines confirmed high uptake in HER2-positive cells, validating specificity and stability. **Conclusions**: The successful synthesis and labelling of the [^89^Zr]Zr-p-NCS-Bz-DFO-anti-HER2 affibody are promising achievements for its further application in targeted immuno-PET imaging for HER2-positive malignancies. Further in vivo studies are needed to support its clinical translation.

## 1. Introduction

Molecular imaging is an indispensable pillar of contemporary biomedical research, enabling high-resolution, non-invasive visualization of biological and in vivo metabolic processes. Among the radioisotopes employed in positron emission tomography (PET), ^89^Zr has gained significant attention due to its favourable characteristics [1]. ^89^Zr undergoes β^+^ decay (22.7%) and electron capture (77%) to ^89m^Y, which subsequently emits high-energy γ-radiation (909 keV), producing a robust PET signal with a high spatial resolution [2,3,4]. Its physical half-life (T_1/2_ = 78.4 h) enables longer time from injection to scanning and makes it possible to perform multiple scans for up to 7–10 days with a single injected dose. These particular advantages recommend ^89^Zr as an ideal candidate for tracking large and slow kinetics biomolecules, such as monoclonal antibodies (mAbs), antibody fragments, and affibodies. Unlike shorter-lived PET isotopes, such as ^18^F (T_1/2_ = 109.7 min) [5], ^61^Cu (T_1/2_ = 3.33 h) [6] or ^68^Ga (T_1/2_ = 67.6 min) [7], which are better suited for fast pharmacokinetics molecules, ^89^Zr facilitates the detailed assessment of antibody biodistribution, tumour retention, metabolic pathways and therapeutic efficacy [2,3]. A critical factor in the success of ^89^Zr-immuno-PET is the choice of bifunctional chelators (BFCs), which ensure stable radiometal complexation without compromising the vector molecule’s biological activity. DFO and its derivatives, such as p-isothiocyanatobenzyl-deferoxamine (p-NCS-Bz-DFO), have been widely employed due to their strong affinity for [^89^Zr]Zr^4+^ and proven in vivo stability [8,9]. However, despite the good thermodynamic stability of the [^89^Zr]Zr -DFO complex, studies have reported in vivo transchelation and bone accumulation of free [^89^Zr]Zr^4+^, raising concerns about long-term biodistribution and off-target effects [10].

To address this challenge, developing and optimizing chelation strategies are required, aiming to enhance the stability of the ^89^Zr complex, while keeping low levels of radiotracer uptake in non-targeted tissues. Clinically, ^89^Zr-immuno-PET has demonstrated significant utility in oncology, particularly for imaging tumours overexpressing human epidermal growth factor receptor 2 (HER2), epidermal growth factor receptor (EGFR) [11], and vascular endothelial growth factor (VEGF) [12,13]. A key application involves the radiolabelling of trastuzumab, a monoclonal antibody targeting HER2-positive breast cancer, where HER2 overexpression drives oncogenic signalling via tyrosine kinase activation [14,15,16]. Given that HER2 overexpression is observed in approx. 20% of breast cancer cases and serves as a critical determinant of prognosis and therapeutic response [17], the refinement of ^89^Zr-based HER2-targeting tracers represents a significant advancement in precision oncology.

**Figure 1:**
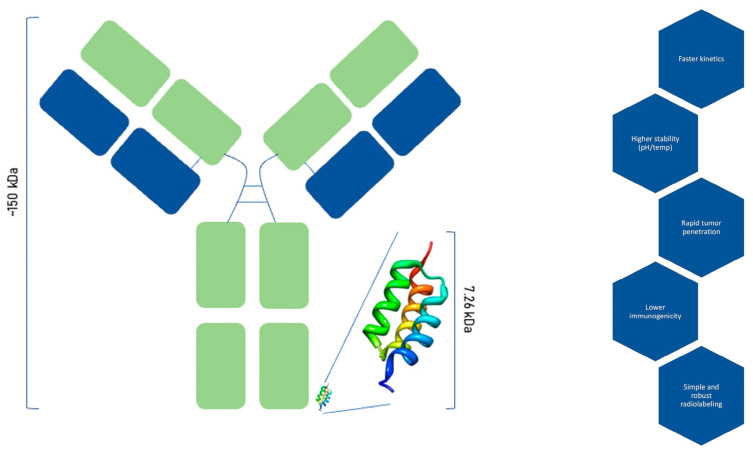
Schematic representation of an affibody molecule, highlighting its small size (approx. 7.5 kDa) and alpha-helical structure, along with the key advantages of affibodies over monoclonal antibodies.

Affibody molecules represent a distinct class of engineered protein scaffolds that offer several advantages over conventional monoclonal antibodies for molecular imaging applications. Structurally, affibodies are composed of a small three-helix complex with a molecular weight of approximately 6.5–7.5 kDa, in contrast to the approx. 150 kDa size of the bigger full-length antibodies. This noticeably reduced size confers superior pharmacokinetics, characterized by rapid blood clearance and enhanced tumour penetration, allowing for high-contrast imaging within a shorter timeframe post-administration (Figure 1). Affibodies consist of 58–63 amino acids, with variations in 13 of these positions generating a diverse library of ligand variants [5,18]. Furthermore, affibodies exhibit remarkable physicochemical stability across a broad range of pH values (2.5–11) and temperatures (up to 90 °C), significantly exceeding the stability profile of antibodies, which are often sensitive to environmental stressors [19,20,21].

From a production standpoint, affibodies can be efficiently expressed in prokaryotic systems such as Escherichia coli, simplifying manufacturing processes and reducing production costs compared to mammalian cell-derived antibodies. Immunogenicity, a critical consideration for repeated administrations, is inherently lower for affibodies owing to their engineered, non-immunoglobulin-based nature. Moreover, the absence of glycosylation and the presence of accessible conjugation sites render affibodies highly amenable to site-specific and robust radiolabelling, facilitating the development of versatile radiopharmaceuticals. These properties position affibodies as highly attractive candidates for targeted imaging modalities, particularly PET, where rapid biodistribution and high tumour penetrability are essential for an optimal imaging contrast [20,21].

While several HER2-binding affibody variants have been radiolabelled using radionuclides such as ^68^Ga, ^64^Cu, ^18^F and ^111^In for molecular imaging applications, radiolabelling with ^89^Zr remains less explored in affibody molecules due to well-documented challenges concerning the stability of the [^89^Zr]Zr-DFO complex under physiological conditions. These challenges include the potential for transchelation and release of free ^89^Zr ions, resulting in undesirable off-target accumulation, especially in bone tissue [22]. The affibody molecule investigated in this study, developed by the Institute of Biochemistry of the Romanian Academy (IBAR), has previously been radiolabelled only with ^52^Mn [18]. In this work, we report the first-ever conjugation of this HER2-targeting affibody with p-NCS-Bz-DFO and its radiolabelling with ^89^Zr. We further assess its in vitro stability and specific uptake in HER2-positive versus HER2-negative cell lines, aiming to validate its potential as a PET imaging agent.

This study aims to optimize the radiolabelling of an anti-HER2 affibody, synthesized and characterized by Prof. Szedlacek’s group from IBAR, using [^89^Zr]Zr-oxalate prepared by our group with ^89^Zr produced by irradiation of ^nat^Y foils at the TR-19 cyclotron (15.2 MeV, 100 μA·h) [23]. The primary objective of the research was to assess the stability and affinity of the resulting ^89^Zr complexes as a PET imaging agent for enhanced diagnosis of HER2-positive tumours. The stability of the [^89^Zr]Zr-p-NCS-Bz-DFO-affibody complex was evaluated for up to 140 h at 37 °C in saline as well as human and rat serum. The receptor affinity of the [^89^Zr]Zr-p-NCS-Bz-DFO-anti-HER2 affibody was subsequently assessed in MCF-7 and BT-474 breast cancer cell lines to prove the utility of the proposed radiopharmaceutical for the accurate and highly specific identification of the HER2-positive tumours.

This work provides the first report of ^89^Zr labelling for this specific HER2-targeting affibody, combining the advantages of rapid tumour targeting with the longer imaging window of ^89^Zr. By focusing on optimizing radiolabelling conditions and evaluating in vitro stability and HER2-specific uptake, our study bridges a current gap in immuno-PET tracer development, offering a promising alternative to full-length antibodies and shorter-lived isotopes.

## 2. Materials and Methods

### 2.1. Chemical Reagents and Equipment

^89^Zr was produced by irradiation of ^nat^Y foils at the TR-19 cyclotron (ACSI- Advanced Cyclotron Systems Inc., Richmond, BC, Canada). The ^nat^Y foils used as targets were purchased from Alfa Aesar (Thermo Fisher Scientific, Karlsruhe, Germany). Hydrochloric acid (HCl, 30%), sodium carbonate (Na_2_CO_3_), human serum, rat serum, and dimethyl sulfoxide (DMSO, >99.9%) were purchased from Sigma-Aldrich (Merck KGaA, Steinheim, Germany). Ultrapure water was produced in-house with the Millipore Direct-8 system (Millipore SAS, Molsheim, France). Nitric acid, oxalic acid, and sodium citrate were purchased from Honeywell Fluka (Honeywell International Inc., Muskegon, MI, USA), and sodium chloride (0.9%) from Hemofarm A.D. (Vršac, Serbia). Methanol and ammonium acetate were supplied by Merck KGaA (Darmstadt, Germany). The p-NCS-Bz-DFO chelator (purity ≥ 95%) used for conjugation was purchased from Macrocyclics Inc. (Dallas, TX, USA). A Zr-specific cartridge from TrisKem International (Bruz, France), loaded with 0.34 g/mL resin, was used for the purification of the [^89^Zr]Zr-oxalate solution. The anti-HER2 affibody was synthesized in-house by our colleagues from the Institute of Biochemistry of the Romanian Academy (IBAR, Bucharest, Romania). An SEC column, Sephadex™ G-25 Medium PD-10, from GE Healthcare (Chicago, IL, USA) was used to purify the immunoconjugates. Radionuclide purity (RNP) and identity were determined by gamma spectrometry, using a high-purity germanium (HPGe) detector from Baltic Scientific Instruments (BSI, Riga, Latvia) operated with the InterWinner software, version 7.0, and by measuring activity on the AtomlabTM 500 dose calibrator from Biodex (New York, NY, USA). The radiochemical purity (RCP) was assessed by radio-thin layer chromatography (radio-TLC) using an ELYSIA-Raytest instrument (Straubenhardt, Germany), equipped with a bismuth germanium oxide (BGO) scintillator detector and a collimator for gamma rays with energies > 450 keV. The GINA X software version 10.4.5.6507 was used for analysis.

The stationary phase used for the TLC method was silica gel on plastic support—plates 60 F254, purchased from Merck KGaA (Darmstadt, Germany). Radio-TLC analyses also involved the use of three mobile phases:

- F1—0.1 M sodium citrate (Carl Roth GmbH, Karlsruhe, Germany);
- F2—mixture of methanol/1 M ammonium acetate (NH_4_OAc), at 7:3 (*v*/*v*) (Merck KGaA, Darmstadt, Germany);
- F3—20 mM citric acid buffered with 1M sodium carbonate, pH 5.0 (Sigma-Aldrich, Steinheim, Germany).

Two human breast cancer cell lines were evaluated: BT-474 (ductal carcinoma, ATCC product, catalogue number HTB-20, sourced from BIO ZYME S.R.L., Cluj-Napoca, Romania) and MCF-7 (adenocarcinoma, ATCC product, catalogue number HTB-22, also from BIO ZYME S.R.L., Cluj-Napoca, Romania) [24,25]. Cells were cultured at 37 °C with 5% CO_2_ in Dulbecco’s modified Eagle medium (DMEM, Gibco, ThermoFisher Scientific, Waltham, MA, USA) supplemented with 10% fetal bovine serum (FBS) (Euroclone, Milan, Italy) and a stabilized antibiotic–antimycotic solution (100×) (Sigma-Aldrich, Saint Louis, MO, USA) containing 10,000 U/mL penicillin, 10 mg/mL streptomycin, and 25 µg/mL amphotericin B. The LigandTracer Yellow equipment with the associated TraceDrawer software (LigandTracer 1.0.1, Ridgeview Instruments, Uppsala, Sweden) was used to measure in vitro uptake and retention on malignant cell lines [26].

### 2.2. Zirconium-89 Production and Radiochemical Processing of the Target

**Figure 2:**
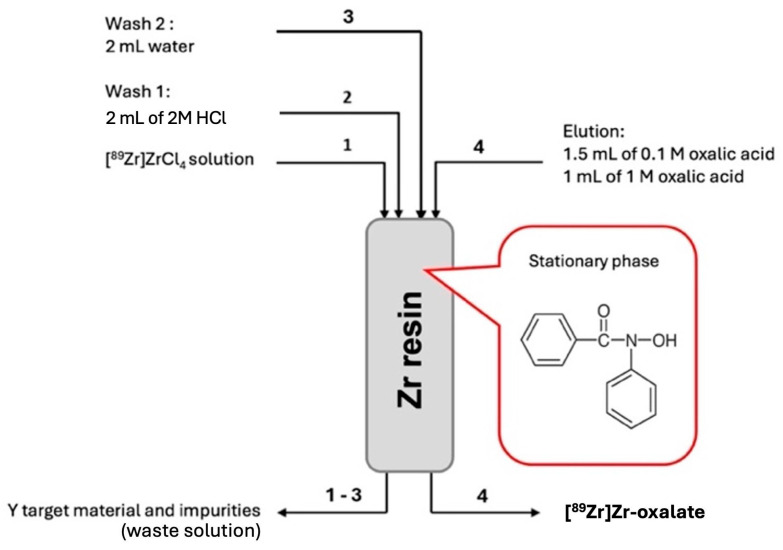
Purification process using the ZR cartridge [27,28].

A ^nat^Y target was bombarded with 12.9 ± 0.78 MeV protons at the TR-19 cyclotron to produce the ^89^Zr radioisotope by ^89^Y(p,n)^89^Zr nuclear reaction. Following irradiation, 2 M and 4 M HCl solutions were used to dissolve the target at 100 °C. A specific ZR cartridge was used to purify the [^89^Zr]ZrCl_4_ solution that resulted after dissolution. Water and 2 M HCl were used to eliminate the impurities from the solution. The final solution was obtained by using 0.1 M and 0.5 M oxalic acid to elute ^89^Zr from the cartridge in the form of [^89^Zr]Zr-oxalate (Figure 2) [27].

### 2.3. Synthesis of Anti-HER2 Affibody

**Figure 3:**
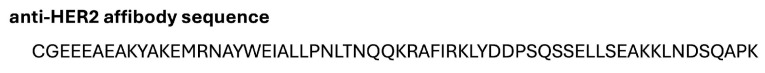
Anti-HER2 affibody sequence [18].

To generate the anti-HER2 fragment, a purification method based on protein expression in E. coli was utilized, with a small ubiquitin-like modifier (SUMO) tag at the N-terminus. The SUMO tag allows specific proteases to cleave the protein without leaving extra residues, resulting in a pure protein with a distinct cysteine residue at the N-terminus. The SUMO-tagged affibody was separated and purified from the cells using sodium dodecyl-sulphate polyacrylamide gel electrophoresis (SDS-PAGE), and the tags were subsequently removed using SUMO protease (Figure 3). After purification, the affibody solution could be used for conjugation with bifunctional chelators. The method of obtaining the affibody was described in detail by our collaborators at IBAR in a previous article, where the same affibody was radiolabelled with Mn-52, investigating a potential positron emission tomography–magnetic resonance imaging (PET-MRI) tracer [18].

### 2.4. Conjugation of p-NCS-Bz-DFO with Anti-HER2 Affibody

**Figure 4:**
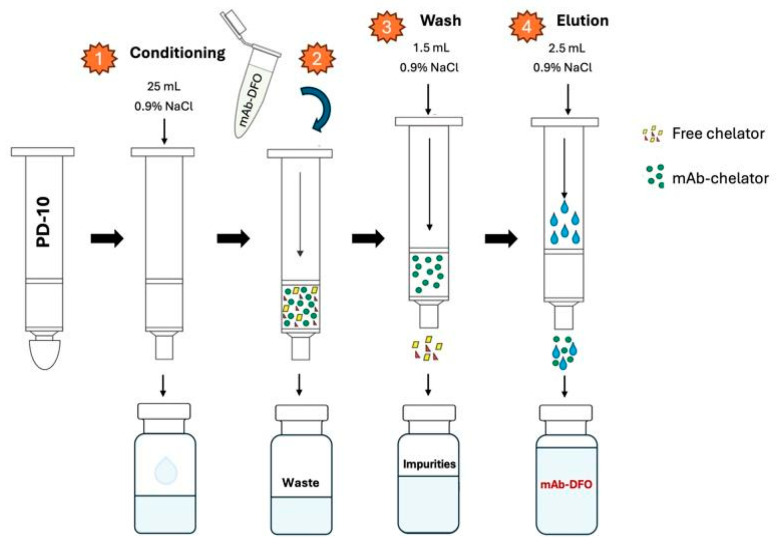
Purification of mAb–chelator conjugate using PD-10 column.

We used 200 µL of anti-HER2 affibody solution (0.3 ± 0.02 mg/mL) and added 12 µL of p-NCS-Bz-DFO solution in DMSO (0.02 mg/µL), with an mAb to chelator mass ratio of 1:4. The pH of the conjugation solution was adjusted to 9.20 ± 0.2, using 0.1 M Na_2_CO_3_ buffer solution. The resulting mixture was incubated at 37 °C for 60 min in a thermomixer (300 rpm). The excess chelator was removed via size exclusion chromatography using a Sephadex™ G-25 Medium PD-10 column and 0.9% NaCl saline. Figure 4 presents the detailed purification process.

### 2.5. [^89^Zr]Zr Radiolabelling of p-NCS-Bz-DFO-Anti-HER2 Affibody Conjugate

**Figure 5:**
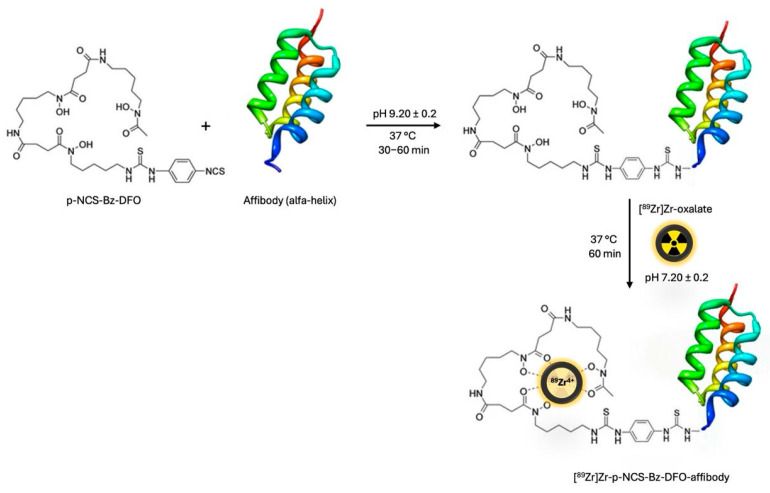
General radiolabelling procedure of [^89^Zr]Zr-NCS-Bz-DFO-affibody [30].

An anti-HER2 affibody, previously conjugated with the bifunctional chelator p-NCS-Bz-DFO, was radiolabelled with [^89^Zr]Zr-oxalate to enable targeting of HER2-overexpressing receptors in breast cancer. The radiolabelling reaction was carried out at 37 °C for 60 min in a thermomixer set to 550 rpm, using 0.5–1.1 mL of [^89^Zr]Zr-oxalate solution, with a radioactive concentration of up to 470 MBq/mL (Figure 5) [29]. The reaction pH was adjusted using 2 M Na_2_CO_3_ to optimize labelling efficiency and systematically varied between 7.0 and 9.0. After incubation, the solution was cooled to room temperature and purified using a PD-10 column [26].

### 2.6. Quality Control Studies of ^89^Zr(IV) Complexes

The pH of the synthesized compounds was evaluated using colorimetric strips. The activity of the [^89^Zr]Zr-oxalate solution was measured using the Biodex dose calibrator. A high-purity germanium (HPGe) coaxial detector, p-type, was employed to identify ^89^Zr and potential radioactive contaminants in the sample (InterWinner software, version 7.0). To achieve an adequate signal-to-noise ratio, the acquisition time was set to 5 min. The test involved analyzing a 2 µL sample to identify the specific peaks of ^89^Zr (511 keV, 909 keV, 1621 keV, 1657 keV, 1713 keV, 1744 keV), as well as the presence of potential radioactive impurities such as ^88^Zr and ^88^Y.

The RCP of the ^89^Zr solutions was assessed using a radio-TLC system. For the [^89^Zr]ZrCl_4_ and [^89^Zr]Zr-oxalate solutions, we used mobile phases consisting of sodium citrate 0.1 M, citric acid 20 mM, and methanol/ammonium acetate 1 M, 7:3 (*v*/*v*), respectively. For the affibody conjugate, the mobile phase sodium citrate 0.1 M was used. We used GINA X software version 10.4.5.6507 for data acquisition and processing. The acquisition time was set to 15 min.

### 2.7. Stability Studies of ^89^Zr(IV) Complexes

The stability of ^89^Zr(IV) complexes was investigated by incubation in solutions of 0.9% saline and human and rat plasma serum, for up to 140 h at 37 °C. The samples were prepared by adding 200 μL of serum to each aliquot of the ^89^Zr complex, with various activity values, ranging from 5.5 to 10 MBq. The RCP of the samples was evaluated using a radio-TLC system.

### 2.8. In Vitro Uptake/Retention Assay

Two human breast cancer cell lines were assessed: BT-474, which presents an overexpression of HER2, and MCF-7, as a negative control cell line for the receptor. The evaluation of the in vitro uptake and retention on the malignant cell lines was performed on the LigandTracer Yellow device.

A sample of 400,000 cells was seeded in a marked, defined area of a Petri dish tilted at a 30° angle. Seeding was performed 24 h before analysis to ensure cell adherence. During data acquisition, the Petri dish was placed on the angled support of the device and periodically rotated to measure radioactivity in two positions: (1) cells in medium with added radioisotope and (2) cells without medium. By calculating the difference between the activities recorded under these two positions, the quantity of radiolabelled substances bound to the cell receptors at regular intervals can be determined. The data acquired is presented graphically, illustrating how the signal intensity varies over time.

## 3. Results

### 3.1. Zirconium-89 Production from Solid Targets and Post-Irradiation Processing

Zirconium-89 was produced at the TR-19 cyclotron (COMECER unit) by irradiating foils of ^nat^Y with a proton beam current of 25 µA, an extracted energy of 15.2 MeV, and an irradiation time of 4 h. In our previous work, we studied the influence of proton beam energy on RNP. Thus, we observed that the highest purity, with minimal contaminant levels of unwanted isotopes like ^88^Zr and ^88^Y, was achieved at a proton beam energy of 12.9 MeV on a target [23]. We dissolved the irradiated target in 2 M and 4 M HCl and then purified the resulting [^89^Zr]ZrCl_4_ solution using an ion exchange resin containing a fenamic acid derivative (R-CO-NH-OH). To eliminate metal impurities, we washed the column with 2 M HCl and water. We eluted ^89^Zr as oxalate using oxalic acid concentrations of 0.1 M and 0.5 M.

Following irradiation and post-irradiation processing, the activity of the [^89^Zr]Zr-oxalate solution was 2.95 ± 0.31 GBq, corrected to the end of bombardment (EOB). Table 1 presents the irradiation parameters used for zirconium-89 production.

### 3.2. Quality Control

**Figure 6:**
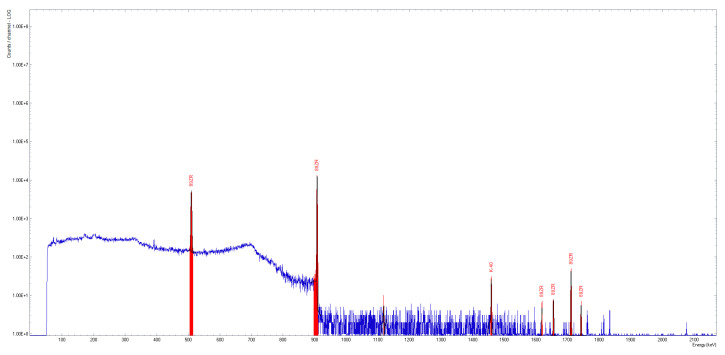
Gamma-ray spectrum of [^89^Zr]Zr-oxalate solution.

The [^89^Zr]Zr-oxalate solution was clear and colourless, with RNP higher than 99.9% (Figure 6).

The analytical quality of the irradiated target during the dissolving and purification processes was assessed using a multi-mobile phase radio-TLC system and a gamma-ray spectrometer. To optimize separation and accurately detect potential colloidal zirconium or zirconium oxide species, we tested three different mobile phases in the radio-TLC analysis. Table 2 presents the retention factor (R_f_) values for each mobile phase, indicating the efficiency of the approach in separating and identifying the final product.

We selected methods 1 and 2 based on the ability of citrate ions to act as a moderate complexing agent compared to stronger agents, such as ethylenediaminetetraacetic acid (EDTA) or diethylenetriamine pentaacetic acid (DTPA). Citrate facilitates differential migration of zirconium species (for the samples at an acidic pH) on the TLC plate, allowing a more precise characterization of impurities. Our results highlight the separation of species using both methods 1 and 2, confirming the applicability of these approaches for impurity evaluation, with RCP exceeding 95%. Notably, using sodium citrate as the mobile phase (method 1) resulted in better separation than the citric acid mobile phase (method 2), as reflected by the distinct R_f_ values for each species. This suggests that sodium citrate provides a more favourable interaction with the stationary phase, enhancing the resolution of zirconium species.

Using method 3 (methanol/ammonium acetate 1 M, 7:3), we observed strong interactions between the sample components and the silica gel stationary phase. This resulted in a single peak, without separation of the zirconium species, indicating that method 3 is unsuitable for impurity analysis and may result in incorrect interpretations of RCP.

RCP remained consistently above 95% for samples with pH values greater than 5 (Supplementary Figures S1–S4).

### 3.3. Assessment of p-NCS-Bz-DFO-Anti-HER2 Affibody Radiolabelling with [^89^Zr]Zr-Oxalate

To optimize radiolabelling efficiency, we systematically adjusted the quantity of anti-HER2 affibody, radioactive concentration of [^89^Zr]Zr-oxalate, pH, and reaction time. The radiolabelling process was structured into three experiments, each varying specific parameters. Using either 6.89 or 8.25 nmoles of anti-HER2 affibody, we applied different volumes of [^89^Zr]Zr-oxalate solution (0.5–1.1 mL), while keeping the temperature (37 °C) and reaction time (1 h) constant. Experiments 1 and 2 kept the pH constant; based on the findings from experiment 2, four different pH levels were introduced in experiment 3. Radiolabelling efficiency and RCP were assessed by radio-TLC (Method 1) and showed significant variation depending on the experimental conditions. Table 3 summarizes the varied parameters and the corresponding outcomes, including radiochemical yield, molar activity and RCP.

**Figure 7:**
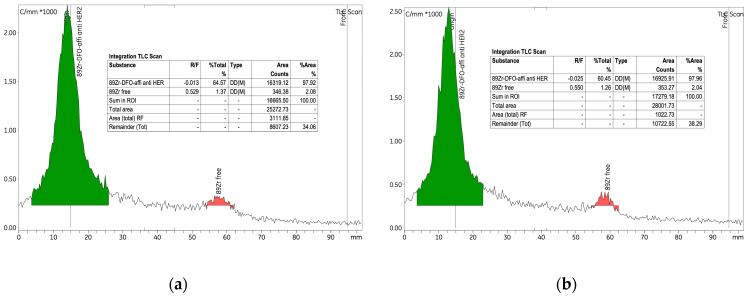
Radio-TLC chromatograms for the [^89^Zr]Zr-p-NCS-Bz-DFO-anti-HER2 affibody complex (experiment 1): (**a**) before purification and (**b**) after purification.

The initial experiment investigated the radiolabelling of the p-NCS-Bz-DFO-anti-HER2 affibody immunoconjugate with ^89^Zr, using 6.89 nmol of anti-HER2 affibody and a radioactive concentration of 0.25 ± 0.06 MBq/μL within the [^89^Zr]Zr-oxalate solution. An amount of 265 nmol of p-NCS-Bz-DFO chelator was used, corresponding to an mAb to chelator mass ratio of 1:4. This experiment established the baseline conditions for the labelling process and provided a reference for the parameter variations introduced in experiments 2 and 3. As detailed in Table 3, the labelling yield was approximately 75%, indicating high efficiency under the experimental conditions. RCP exceeded 97% both before and after purification, with the amount of free ^89^Zr consistently low, at approx. 2% (Figure 7).

In experiment 2, we investigated the impact of increasing the quantity of anti-HER2 affibody on the radiolabelling process, while keeping other parameters constant and optimizing certain conditions to achieve the highest reaction yield and RCP. In the context of radiolabelling affibody molecules with [^89^Zr]Zr-oxalate, an unfavourable ratio between the radioactivity concentration and the amount of affibody can significantly impact the efficiency and quality of the final radioconjugate. This imbalance is typically characterized by a high radioactivity concentration relative to a low affibody mass, which leads to several challenges: reduced labelling efficiency, precipitation, suboptimal chelation and biodistribution issues. To avoid these issues, it is essential to optimize the molar ratio between ^89^Zr and the affibody.

**Figure 8:**
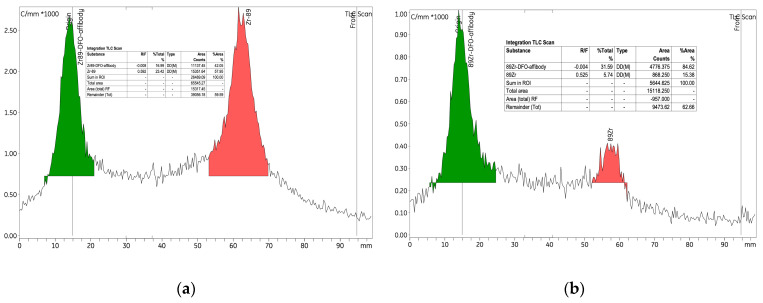
Radio-TLC chromatograms of the [^89^Zr]Zr-p-NCS-Bz-DFO-anti-HER2 affibody solution from experiment 2 after (**a**) 1 h from the initiation of the reaction and (**b**) 12 h at 37 °C.

To label 8.25 nmol of anti-HER2 affibody, we used a radioactive concentration of 0.44 MBq/μL of [^89^Zr]Zr-oxalate solution. Additionally, the quantity of p-NCS-Bz-DFO was increased to 318.8 nmol to maintain the same 1:4 ratio (mAb/chelator). After 12 h from the start of the labelling, the yield increased from 34% to approximately 68%, indicating a value comparable to experiment 1 (labelling yield 75%). Given the extended reaction time (up to 12 h), this suggests a relatively low labelling rate efficiency. The RCP of the [^89^Zr]Zr-p-NCS-Bz-DFO–anti-HER2 affibody complex improved significantly from 42% to 84.62% (Figure 8). When comparing these results with those from experiments 1 and 3, which used similar conditions, we observed a decrease in RCP, potentially due to an unfavourable ratio between anti-HER2 affibody quantity and the radioactive concentration of the [^89^Zr]Zr-oxalate solution. This decrease in efficiency highlighted the need to adjust the radioactive concentration of the [^89^Zr]Zr-oxalate solution used in the reaction, to ensure a high radiochemical yield while preserving the biological functionality of the affibody.

In experiment 3, we studied the influence of pH variation (7.0–9.0) on the radiolabelling efficiency of the anti-HER2 affibody using [^89^Zr]Zr-oxalate solution. As summarized in Table 3, labelling yield strongly depended on pH, reaching a maximum of 78.41% at pH 7.4. Increasing the pH to more alkaline values caused a progressive decrease in labelling efficiency, with the lowest yield of 11.96% recorded at pH 9.0.

**Figure 9:**
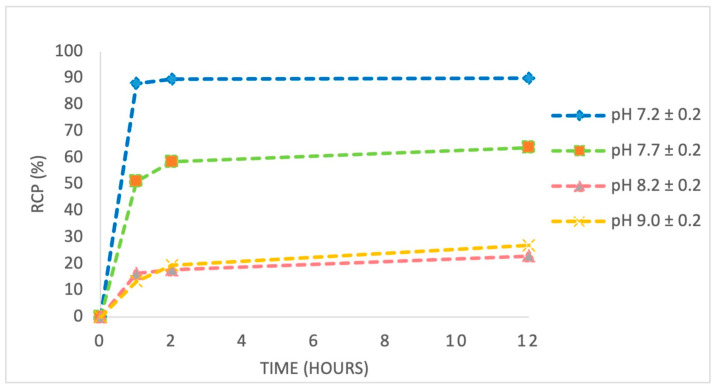
Influence of labelling pH on radiochemical purity over time.

We monitored the evolution of the radiolabelling process via radio-TLC at 1, 2, and 12 h intervals (Supplementary Figures S5–S7). Figure 9 indicates that, at pH 7.0–7.5, RCP remained stable above 97% for at least 12 h, confirming the robustness of the complex under physiological conditions. In contrast, RCP significantly decreased at alkaline pH values (8.0–9.0), indicating a slowed labelling reaction and degradation of the complex.

**Figure 10:**
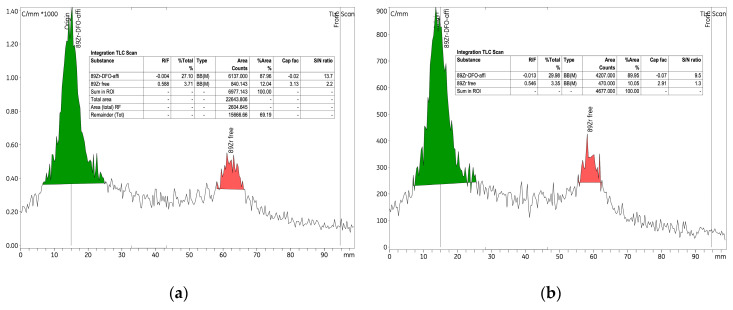
Radio-TLC chromatograms for the [^89^Zr]Zr-p-NCS-Bz-DFO-anti-HER2 affibody complex from experiment 3 for the pH labelling of 7.0–7.5 (**a**) after 1 h from the initiation of the reaction and (**b**) after 12 h at 37 °C.

The observed decrease in labelling efficiency and complex stability at higher pH values is likely due to enhanced hydrolysis of [^89^Zr]Zr-oxalate, which promotes the formation of sparingly soluble zirconium hydroxide species (e.g., [^89^Zr]Zr(OH)_4_) that exhibit reduced reactivity toward chelation. Additionally, the elevated hydroxide ion concentration at alkaline pH may interfere with the coordination process by competing with the p-NCS-Bz-DFO chelator for [^89^Zr]Zr^4+^ ions, thereby reducing the efficiency of complex formation. These findings underscore the critical importance of maintaining pH near 7.4 to ensure optimal stability and efficiency in the radiolabelling process (Figure 10).

Alkaline conditions impair chelation efficiency and promote degradation pathways, leading to reduced RCP and potentially compromising the biological performance of the labelled affibody. Furthermore, careful adjustment of experimental parameters—particularly pH and radioactivity concentration—significantly improves both labelling efficiency and radiochemical stability. These optimizations are essential not only for achieving consistent in vitro results but also for ensuring robust in vivo performance, where complex stability, favourable biodistribution, and target specificity are critical for effective molecular imaging.

### 3.4. Stability of [^89^Zr]Zr-Oxalate and [^89^Zr]Zr-p-NCS-Bz-DFO-Anti-HER2 Affibody

We evaluated the stability of [^89^Zr]Zr-oxalate and [^89^Zr]Zr-p-NCS-Bz-DFO in saline and human and rat serum for up to 72 h. For the [^89^Zr]Zr-p-NCS-Bz-DFO-anti-HER2 affibody complex, we extended the monitoring period to 140 h, despite its short plasma half-life (30–60 min), due to its rapid renal clearance and potential clinical relevance.

**Figure 11:**
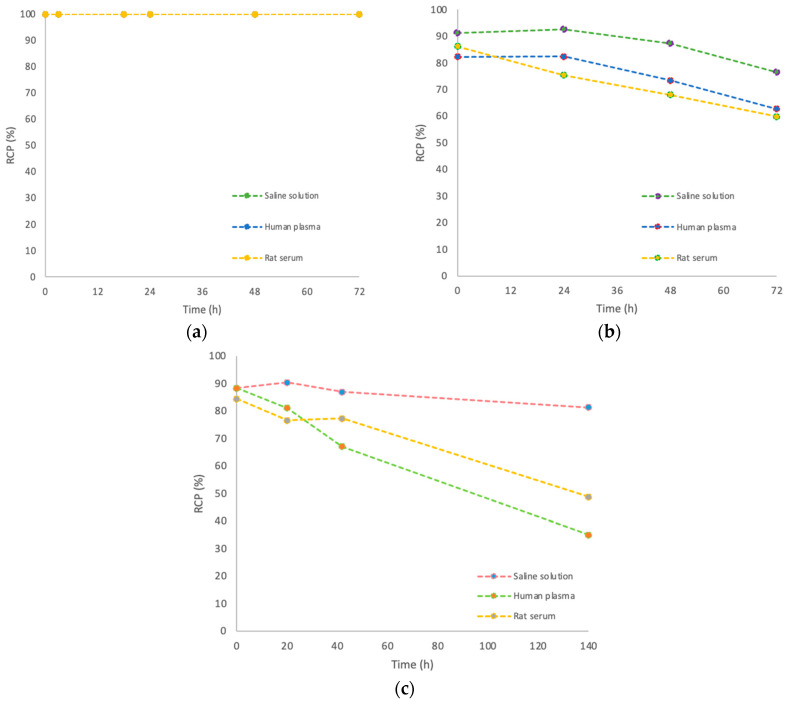
Stability of [^89^Zr]Zr-oxalate (**a**), [^89^Zr]Zr-p-NCS-Bz-DFO (**b**), and (**c**) [^89^Zr]Zr-p-NCS-Bz-DFO–anti-HER2 affibody in saline solution and human and rat serum.

As shown in Figure 11, the [^89^Zr]Zr-oxalate solution remained stable under the test conditions, confirming its suitability as a radiolabelling precursor. In contrast, the [^89^Zr]Zr-p-NCS-Bz-DFO complex presented a progressive decrease in RCP after 72 h of incubation—14.73% in saline solution, 19.54% in human serum and 26.41% in rat serum.

The RCP of the [^89^Zr]Zr-p-NCS-Bz-DFO-anti-HER2 affibody exhibited a more significant decrease, particularly in biological medium, with the degradation beginning as early as 24 h. After 140 h of incubation, the RCP decreased by 7.1% in saline solution, while dropping to 53.42% in human serum and 35.63% in rat serum, respectively. This accelerated degradation in serum may be caused by proteolytic enzymes or transchelation processes. Overall, these stability data underscore the importance of considering the biological environment’s effects on the radioconjugate, which may impact its in vivo performance and imaging efficacy.

### 3.5. Cell Uptake Experiment

Three different solutions of [^89^Zr]Zr-p-NCS-Bz-DFO-affibody each with varying RCPs were compared. The goal was to observe the influence of RCP on cellular retention in the HER2-positive BT-474 cell line. Given the long half-life of the radioisotope (78.41 h), a 2 h uptake assessment was established for real-time ligand–receptor interaction analysis, followed by a minimum of eight data points for a retention assay until a plateau was observed.

Between the uptake and retention, the culture medium with free [^89^Zr]Zr-p-NCS-Bz-DFO-affibody was removed and the cells were washed with fresh, prewarmed culture medium, which was then replaced in the Petri dish.

**Figure 12:**
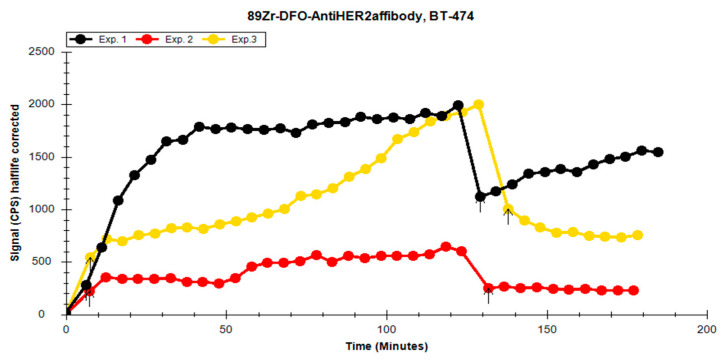
Overlay of the uptake/retention curves of the [^89^Zr]Zr-p-NCS-Bz-DFO-anti-HER2 affibody from the 3 experiments in BT-474. Arrows indicate the first point of the uptake and the retention phase, respectively.

During the experiment, we observed differences between the uptake and retention profiles on the HER2-positive cell line dependent on the RCP of the solution used (Figure 12). In experiments 1 and 3, which had higher RCPs compared to experiment 2, the high affinity of the radiolabelled affibody for the cell line was noticeable. Following the washing procedures before the retention phase, the levels of the affibody bound to the cell receptors remained elevated, suggesting a strong interaction between the affibody and the receptors. In the case of experiment 2, which used a solution with lower RCP, it resulted in reduced uptake in the cell line.

Based on the results obtained, we identified the solution with the highest labelling yield and proper RCP, which demonstrated the strongest interaction with the receptors on the tumour cells. The high labelling yield implies a reduced amount of “cold” affibody in the final solution, ensuring that the tumour cell receptors are predominantly blocked by the Zr-labelled affibody. This solution (experiment 3) was then selected for comparison with an HER2-negative cell line to assess the specificity of the radiolabelled compound.

**Figure 13:**
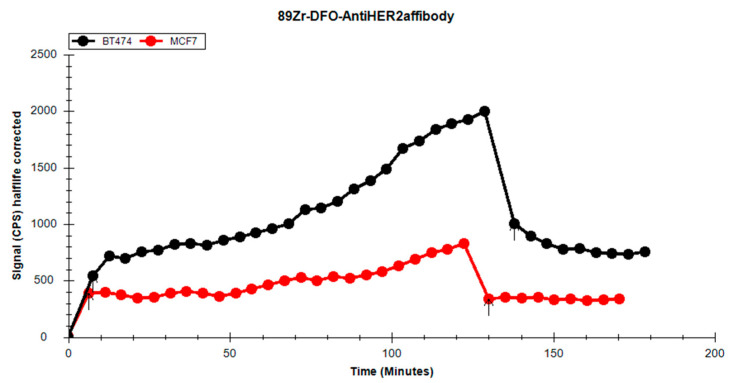
Overlay of the uptake/retention curves of the [^89^Zr]Zr-p-NCS-Bz-DFO-anti-HER2 affibody in BT-474 and MCF-7.

When comparing the uptake between the HER2-positive and HER2-negative cell lines (Figure 13), the BT-474 cell line exhibited more consistent uptake over time. In the retention phase of the analysis, a substantial decrease in activity for the MCF-7 cell line was observed, falling below the initial uptake level, indicating a weaker interaction between cells and the radioligand. The BT-474 cell line demonstrated better retention; the washing procedure removed only about half of the radioactivity, with the remaining activity stably retained by the cells.

We performed a statistical analysis using the *t*-test for the uptake/retention curves and determined that they differed significantly from one another, both globally and within the uptake and retention phases (*p* < 0.005, t stat = 7.396175041, df = 41).

## 4. Discussion

The findings of this study support the potential of a ^89^Zr-labelled anti-HER2 affibody as a promising alternative to conventional monoclonal antibodies for PET imaging of HER2-positive tumours. Although ^89^Zr-trastuzumab and ^89^Zr-pertuzumab have demonstrated clinical efficacy, their utility is limited by extended systemic half-lives, delayed imaging windows (3–5 days post-injection), and increased radiation dose to healthy tissues [2,31,32,33]. Affibody molecules offer several intrinsic advantages that address these limitations, notably rapid systemic clearance, high tumour-to-background ratios shortly after injection, and effective tissue penetration due to their small size (approx. 7.5 kDa).

Our work revealed that the [^89^Zr]Zr-oxalate solution, which we obtained after processing an irradiated foil of ^nat^Y at the TR-19 cyclotron, meets and exceeds the rigorous standards set by the European Pharmacopoeia for RNP (99.9%) and RCP (>95%). Maintaining those parameters plays an important role in the labelling process of the anti-HER2 affibody. Values below the established reference could introduce undesired radioisotopes in the final product. Such contamination compromises the accuracy of PET imaging and increases risks for patients.

A significant finding of this study is the superior stability of our ^89^Zr-labelled affibody at 24 h, with an RCP of over 75% in both human and rat serum. This contrasts with previous reports describing lower serum stability for similar complexes. For instance, ref. [34] reported an RCP of approximately 65% after 24 h for [^89^Zr]Zr-DFO-2Rs15d in human serum. Likewise, ref. [35] observed faster degradation of ^89^Zr-labelled affibody molecules under comparable conditions. This difference suggests improved structural integrity of the radioconjugate under physiological conditions, potentially due to optimized chelation chemistry, enhanced conformational shielding of the radiometal complex, or increased steric protection conferred by the affibody scaffold.

The labelling results showed that the balance between the radioactive concentration and the quantity of anti-HER2 affibody had a significant impact on the reaction’s efficiency. Using a radioactive concentration of 0.44 MBq/µL to label 8.25 nmol of anti-HER2 affibody, we obtained a yield of 33.83% with an RCP of 42.05%. Reducing the concentration to 0.22 ± 0.02 MBq/µL increased the yield by 44.17%, reaching 78%, and improved RCP to 87.96%.

We also demonstrated that pH strongly affects the efficiency of the labelling reaction. The [^89^Zr]Zr-p-NCS-Bz-DFO-anti-HER2 construct developed in this study achieved a radiochemical yield > 75% and an RCP exceeding 87% under near-physiological labelling conditions (pH 7.2 ± 0.2), supporting the robustness and efficiency of the labelling protocol. These values are in line with, or superior to, those reported for similar constructs, particularly under near-physiological pH conditions, as demonstrated in the literature [22,36].

In our stability analysis of [^89^Zr]Zr(IV) complexes in saline and rat and human serum, we confirmed that [^89^Zr]Zr-oxalate remains stable in all solutions. However, we observed degradation of [^89^Zr]Zr-p-NCS-Bz-DFO-anti-HER2 affibody in human serum after 24 h of incubation. Stability in serum is especially significant because it shows that the complex can circulate long enough in the bloodstream to accumulate in tumour tissues, enabling high-resolution PET imaging.

To further improve the serum stability of the complexes, future studies could investigate the use of more stable zirconium chelators, such as DFO*, DFO-cyclo* or other macrocyclic ligands, which have demonstrated higher stability in biological medium [22,37,38]. Additionally, implementing site-specific conjugation strategies may limit the exposure of the chelator to enzymatic degradation [39,40]. Another possible approach could be click chemistry or enzymatic ligation, combined with hydrophilic spacers or albumin-binding domains, to enhance structural shielding and favourable pharmacokinetics [41,42,43,44].

The decreased activity in the MCF-7 cell line, when compared to BT-474, is attributed to differences in receptor expression on their respective cell surfaces. According to the literature [45,46], MCF-7 is an HER-negative cell line. The activity retained on the cells during incubation is due to non-specific binding of p-NCS-Bz-DFO-anti-HER2 to other structures on the cell surface. During the retention phase, characterized by the removal of the radioactive culture medium and the addition of fresh medium, we observed a sharp drop in activity. The retention curve for MCF-7 quickly stabilized, forming a plateau, with the value of the initial retention point already lower than the first uptake point.

In contrast, BT-474 cells displayed significantly higher activity uptake. During the retention phase, we observed two key differences compared to MCF-7: the initial point of the retention curve lies approximately midway through the uptake curve, and the retention curve shows a downward trend from the start. The curvature indicates a stronger retention of the p-NCS-Bz-DFO-anti-HER2 by BT-474 cells. The gradual decline in activity over time is likely due to fresh medium being added. During analysis, the fresh medium washes over the cells, facilitating redistribution of the radiolabelled affibody and leading to equilibrium between the medium and the cells, indicated by the plateauing trend towards the end of the analysis.

## 5. Conclusions

We produced zirconium-89 at the TR-19 cyclotron by irradiating a ^nat^Y target via (p,n) nuclear reaction, with yield and purity proper for the subsequent processing and radiolabelling. By manual processing, we prepared [^89^Zr]Zr-oxalate solution with RNP >99.9% and RCP higher than 95%, complying with the acceptance criteria of the European Pharmacopoeia. This process yielded an EOB-corrected activity of 2.95 ± 0.31 GBq/batch, and the resulting solution was suitable for labelling the p-NCS-Bz-DFO-anti-HER2 affibody immunoconjugate.

We demonstrated that labelling efficiency and RCP were strongly influenced by the ratio between the amount of zirconium (quantitatively correlated with the radioactive concentration) and the quantity of anti-HER2 affibody; an excess of zirconium negatively impacted both parameters. Maintaining a physiological pH (7.2 ± 0.2) during the labelling reaction proved critical for achieving RCP levels above 85%, while deviations from this range significantly reduced complexation efficiency.

Stability studies confirmed that [^89^Zr]Zr-oxalate remained stable in all tested mediums, whereas the [^89^Zr]Zr-p-NCS-Bz-DFO-anti-HER2 affibody showed a gradual decrease in RCP over time, particularly in serum after 24 h.

In vitro binding studies demonstrated selective affinity, as well as a good uptake and retention, of the radiolabelled affibody in the HER2-positive BT-474, supporting its potential for targeted tumour imaging. Based on these promising results, we plan to investigate the in vivo behaviour and tumour-targeting efficacy of the [^89^Zr]Zr-p-NCS-Bz-DFO-anti-HER2 affibody in xenograft mouse models. Due to its small size and rapid clearance, the affibody structure may offer advantages over full-length antibodies for high-resolution PET imaging of HER2-positive tumours.

These findings recommend the [^89^Zr]Zr-p-NCS-Bz-DFO-anti-HER2 affibody as a promising radiotracer for PET imaging. Nevertheless, further comprehensive in vivo studies are needed to fully assess its pharmacokinetics, dosimetry, and safety profile. The favourable biodistribution characteristics of the affibody support its potential clinical translation, helping to improve imaging contrast and reduce radiation burden compared to full-length antibodies.

## Figures and Tables

**Table 1:** Irradiation parameters used for ^89^Zr production.

Parameters	Value
Proton energy on target (MeV)	12.9 ± 0.78
Beam current (μA)	25
Integrated beam current (μA·h)	100
Irradiation time (hours)	4
Target Activity * (GBq)	2.95 ± 0.31
Production efficacy (MBq/μA)	118 ± 12.4
Production yield (MBq/μA·h)	29.5 ± 3.1

* Activity corrected to EOB.

**Table 2:** Resulting R_f_ values for each mobile phase during the monitoring process.

	Value R_f_				
Developing Bath	Method 1 (0.1 M Sodium Citrate)		Method 2 (20 mM Citric Acid)		Method 3 (Methanol/NH_4_OAc 1M, 7:3 *v*/*v*)
[^89^Zr]ZrCl_4_ (2 M + 4 M HCl)	Peak 1 0.021	Peak 2 0.713	Peak 1 0.017	Peak 2 0.363	0.008
[^89^Zr]Zr-oxalate (pH 1.5)	Peak 1 0.038	Peak 2 0.604	Peak 1 0.004	Peak 2 0.338	0.018
[^89^Zr]Zr-oxalate (pH 5.0)	0.629		0.196		0.163
[^89^Zr]Zr-oxalate (pH 7.0)	0.588		0.246		0.004

**Table 3:** Radiolabelling conditions for p-NCS-Bz-DFO-anti-HER2 affibody with [^89^Zr]Zr-oxalate solution. Radiochemical yield and molar activity determined by radio-TLC.

Parameters	Experiment 1	Experiment 2	Experiment 3			
			a	b	c	d
Number of experiments (N)	3	1	1	1	1	1
Quantity of affibody (nmol)	6.89	8.25	8.25			
Quantity of p-NCS-Bz-DFO (nmol)	265.0	318.8	318.8			
pH labelling	7.2 ± 0.2	7.2 ± 0.2	7.2 ± 0.2	7.7 ± 0.2	8.2 ± 0.2	9.0 ± 0.2
[^89^Zr]Zr-oxalate activity (MBq)	265.5 ± 91.2	460.0	112.1 ± 8.2			
Temperature (°C)	37	37	37			
Reaction time (min)	60	60	60			
Radiochemical yield (%)	75.09 ± 7.2	33.83	78.41	45.45	14.59	11.96
Molar activity (MBq/nmol)	26.5 ± 4.4	18.86	11.45	5.97	2.07	1.49
RCP (%)	97.96	42.05	87.96	50.99	16.35	13.41

## Data Availability

The original contributions presented in this study are included in the article/supplementary material. Further inquiries can be directed to the corresponding authors.

## Supplementary Materials

The following supporting information can be downloaded at https://www.mdpi.com/article/10.3390/pharmaceutics17060739/s1: Figure S1: Radio-TLC chromatograms for the [^89^Zr]ZrCl_4_ solution (target dissolved in HCl) using the three mobile phases: (A) 0.1M sodium citrate (method 1), (B) 20 mM citric acid (method 2), (C) methanol/ammonium acetate 1M 7:3 (method 3). Figure S2: Radio-TLC chromatograms for the [^89^Zr]Zr-oxalate (1.5 pH) solution using the three mobile phases: (A) 0.1M sodium citrate (method 1), (B) 20 mM citric acid (method 2), (C) methanol/ammonium acetate 1M 7:3 (method 3). Figure S3: Radio-TLC chromatograms for the [^89^Zr]Zr-oxalate (5.0 pH) solution using the three mobile phases: (A) 0.1M sodium citrate (method 1), (B) 20 mM citric acid (method 2), (C) methanol/ammonium acetate 1M 7:3 (method 3). Figure S4: Radio-TLC chromatograms for the [^89^Zr]Zr-oxalate (7.0 pH) solution using the three mobile phases: (A) 0.1 M sodium citrate (method 1), (B) 20 mM citric acid (method 2), (C) methanol/ammonium acetate 1M 7:3 (method 3). Figure S5: Radio TLC chromatograms obtained after (A) 1 h and (B) 12 h, respectively, following the analysis of the [^89^Zr]Zr-p-NCS-Bz-DFO-anti-HER2 affibody solution labelled at 7.5–8.0 pH (experiment 3). Figure S6: Radio TLC chromatograms obtained after (A) 1 h and (B) 12 h, respectively, following the analysis of the [^89^Zr]Zr-p-NCS-Bz-DFO-anti-HER2 affibody solution labelled at 8.0–8.5 pH (experiment 3). Figure S7: Radio TLC chromatograms obtained after (A) 1 h and (B) 12 h, respectively, following the analysis of the [^89^Zr]Zr-p-NCS-Bz-DFO-anti-HER2 affibody solution labelled at 9.0 pH (experiment 3).

## Abbreviations

BFC	bifunctional chelating agent
BGO	bismuth germanium oxide
BT-474	metastatic human breast ductal carcinoma cell line
DFO	deferoxamine
DMEM	Dulbecco’s modified Eagle medium
DMSO	dimethyl sulfoxide
DTPA	diethylenetriamine pentaacetic acid
EDTA	ethylenediaminetetraacetic acid
EGFR	epidermal growth factor receptor
EOB	end of bombardment
HER2	epidermal growth factor receptor 2
HPGe	high-purity germanium
IBAR	Institute of Biochemistry of the Romanian Academy
MCF-7	Michigan Cancer Foundation-7
MRI	magnetic resonance imaging
Na_2_CO_3_	sodium carbonate
NH_4_OAc	ammonium acetate
p-NCS-Bz-DFO	p-isothiocyanatobenzyl-deferoxamine
PET	positron emission tomography
RNP	radionuclide purity
RCP	radiochemical purity
Rf	retention factor
SDS-PAGE	sodium dodecyl-sulphate polyacrylamide gel electrophoresis
TLC	Thin-layer chromatography
VEGF	vascular endothelial growth factor
