# Supplementary material for: 89Zr-Radiolabelling of p-NCS-Bz-DFO-Anti-HER2 Affibody Immunoconjugate: Characterization and Assessment of In Vitro Potential in HER2-Positive Breast Cancer Imaging
Source: Pharmaceutics. 2025 Jun 4;17(6):739. doi: 10.3390/pharmaceutics17060739 (PMC12196019; doi:10.3390/pharmaceutics17060739)
Supplement: Supplementary file 1 [file pharmaceutics-17-00739-s001.zip › pharmaceutics-3656265-supplementary.pdf]

## Supplementary Materials

# $^{89}$Zr-radiolabelling of p-NCS-Bz-DFO-anti-HER2 affibody immunoconjugate: Characterization and assessment of *in vitro* potential in HER2-positive breast cancer imaging

**Maria-Roxana Tudoroiu-Cornoiu[1, 2], Radu Marian Șerban[1], Diana Cocioabă[1, *], Dragoș Andrei Niculae[1,3, *], Doina Drăgănescu[3], Radu Leonte[1], Alina Catrinel Ion[2], and Dana Niculae[1, 3, *]**

1 Radiopharmaceutical Research Centre (CCR), Horia Hulubei National Institute for R&D for Physics and Nuclear Engineering (IFIN-HH), 30 Reactorului Street, 077125 Magurele, Romania; roxana.cornoiu@nipne.ro, radu.serban@nipne.ro, diana.cocioaba@nipne.ro, radu.leonte@nipne.ro, dana.niculae@nipne.ro
2 Faculty of Chemical Engineering and Biotechnologies, Doctoral School of Applied Chemistry and Materials Science, National University of Science and Technology Politehnica Bucharest, 1-7 Gheorghe Polizu Street, 011061 Bucharest, Romania; ac_ion@yahoo.com
3 Faculty of Pharmacy, Carol Davila University of Medicine and Pharmacy, 6 Traian Vuia Street, 020956 Bucharest, Romania; farmdragosniculae@gmail.com, doina.draganescu@umfcd.ro
* Correspondence: dana.niculae@nipne.ro, diana.cocioaba@nipne.ro, farmdragosniculae@gmail.com

**(A)**

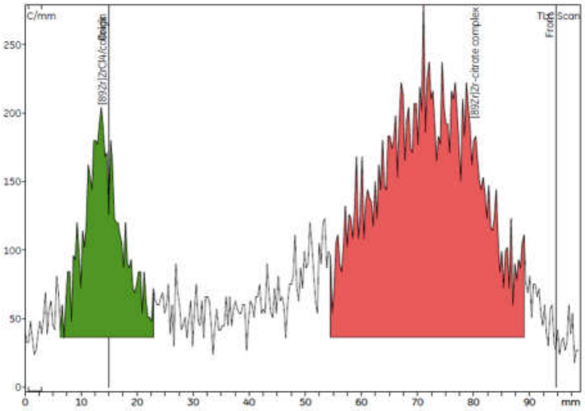


**Integration TLC Scan**

| Substance | R/F | %Total % | Type | Area Counts | %Area % | Cap fac | S/N ratio |
|---|---|---|---|---|---|---|---|
| [89Zr]ZrCl4/colloids | -0.021 | 12.09 | DD(M) | 1196.857 | 22.69 | -0.11 | 4.7 |
| [89Zr]Zr-citrate complex | 0.713 | 41.17 | DD(M) | 4077.143 | 77.31 | 3.80 | 4.6 |
| Sum in ROI | - | - | - | 5274.000 | 100.00 | - | - |
| Total area | - | - | - | 9903.285 | - | - | - |
| Area (total) RF | - | - | - | 8469.000 | - | - | - |

**(B)**

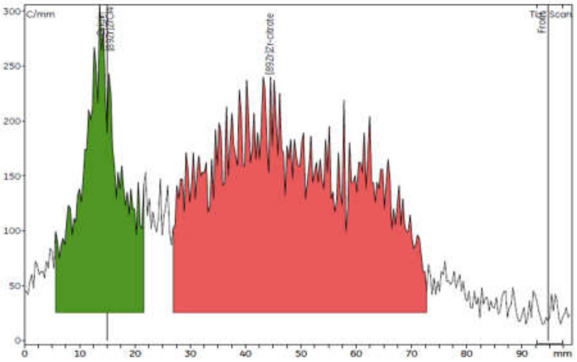


**Integration TLC Scan**

| Substance | R/F | %Total % | Type | Area Counts | %Area % | Cap fac | S/N ratio |
|---|---|---|---|---|---|---|---|
| [89Zr]ZrCl4 | 0.017 | 22.81 | DD(M) | 2067.000 | 25.96 | -0.09 | 7.2 |
| [89Zr]Zr-citrate | 0.363 | 65.04 | DD(M) | 5895.000 | 74.04 | 1.93 | 4.9 |
| Sum in ROI | - | - | - | 7962.000 | 100.00 | - | - |
| Total area | - | - | - | 9063.499 | - | - | - |
| Area (total) RF | - | - | - | 7476.000 | - | - | - |

**(C)**

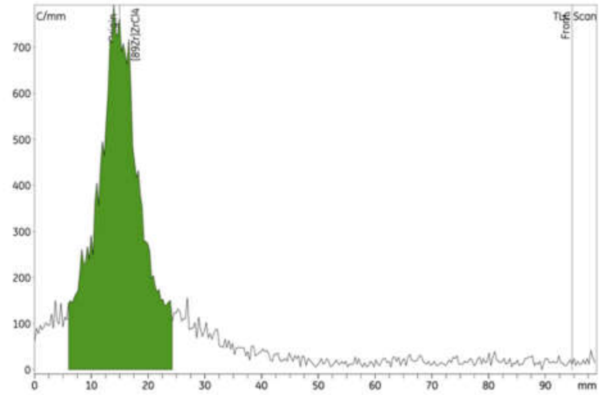


**Integration TLC Scan**

| Substance | R/F | %Total % | Type | Area Counts | %Area % |
|---|---|---|---|---|---|
| [89Zr]ZrCl4 | 0.008 | 70.53 | DD(M) | 6835.000 | 100.00 |
| Sum in ROI | - | - | - | 6835.000 | 100.00 |
| Total area | - | - | - | 9691.000 | - |
| Area (total) RF | - | - | - | 5586.000 | - |
| - | - | - | - | - | - |

**Figure S1.** Radio-TLC chromatograms for the [$^{89}$Zr]$ZrCl_4$ solution (target dissolved in HCl) using the three mobile phases: (A) 0.1M sodium citrate (method 1), (B) 20 mM citric acid (method 2), (C) methanol: ammonium acetate 1M= 7:3 (method 3)

**(A)**

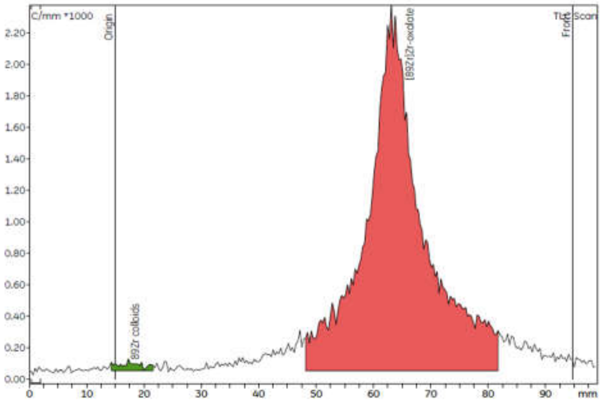


**Integration TLC Scan**

| Substance | R/F | %Total % | Type | Area Counts | %Area % | Cap fac | S/N ratio |
|---|---|---|---|---|---|---|---|
| 89Zr colloids | 0.038 | 0.79 | DD(M) | 276.60 | 1.07 | 0.20 | 0.0 |
| [89Zr]Zr-oxalate | 0.604 | 73.28 | DD(M) | 25666.80 | 98.93 | 3.22 | 21.8 |
| Sum in ROI | - | - | - | 25943.40 | 100.00 | - | - |
| Total area | - | - | - | 35024.40 | - | - | - |
| Area (total) RF | - | - | - | 33688.00 | - | - | - |

**(B)**

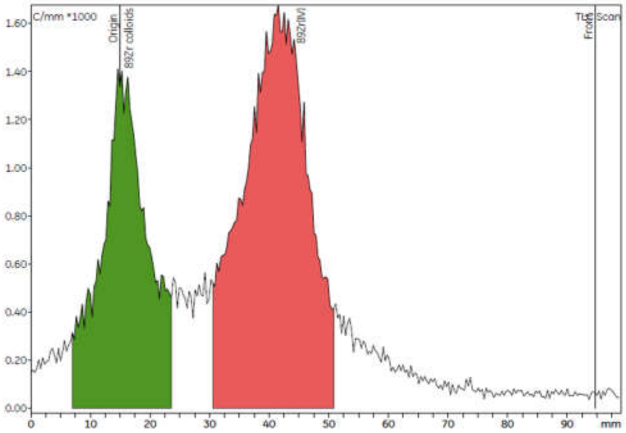


**Integration TLC Scan**

| Substance | R/F | %Total % | Type | Area Counts | %Area % | Cap fac | S/N ratio |
|---|---|---|---|---|---|---|---|
| 89Zr colloids | 0.004 | 27.97 | DD(M) | 12385.00 | 36.94 | 0.02 | 13.9 |
| 89Zr(IV) | 0.338 | 47.74 | DD(M) | 21140.00 | 63.06 | 1.80 | 11.3 |
| Sum in ROI | - | - | - | 33525.00 | 100.00 | - | - |
| Total area | - | - | - | 44280.00 | - | - | - |
| Area (total) RF | - | - | - | 37513.00 | - | - | - |

**(C)**

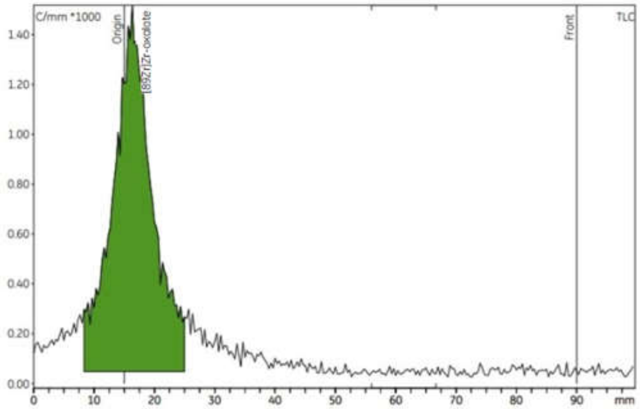


**Integration TLC**

| Substance | R/F | %Total % | Type | Area Counts | %Area % |
|---|---|---|---|---|---|
| [89Zr]Zr-oxalate | 0.018 | 69.40 | DD(M) | 10894.25 | 100.00 |
| Sum in ROI | - | - | - | 10894.25 | 100.00 |
| Total area | - | - | - | 15698.88 | - |
| Area (total) RF | - | - | - | 9246.13 | - |
| Remainder (Tot) | - | - | - | 4804.63 | 30.60 |

**Figure S2.** Radio-TLC chromatograms for the [$^{89}$Zr]Zr-oxalate (1.5 pH) solution using the three mobile phases: (A) 0.1M sodium citrate (method 1), (B) 20 mM citric acid (method 2), (C) methanol: ammonium acetate 1M= 7:3 (method 3)

**(A)**

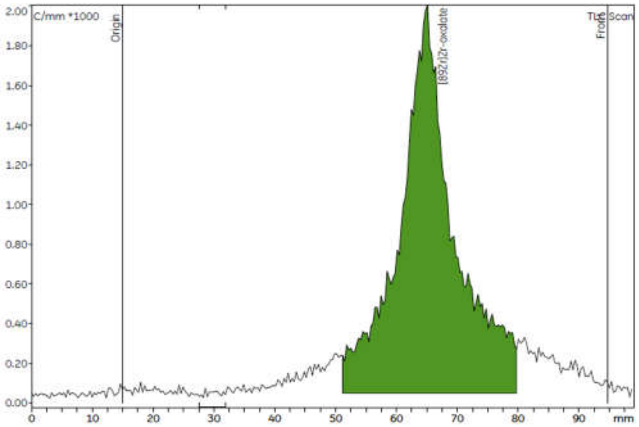


**Integration TLC Scan**

| Substance | R/F | %Total % | Type | Area Counts | %Area % | Cap fac | S/N ratio |
|---|---|---|---|---|---|---|---|
| [89Zr]Zr-oxalate | 0.629 | 73.39 | DD(M) | 20127.15 | 100.00 | 3.36 | 24.2 |
| Sum in ROI | - | - | - | 20127.15 | 100.00 | - | - |
| Total area | - | - | - | 27425.46 | - | - | - |
| Area (total) RF | - | - | - | 23953.62 | - | - | - |
| Remainder (Tot) | - | - | - | 7298.31 | 26.61 | - | - |

**(B)**

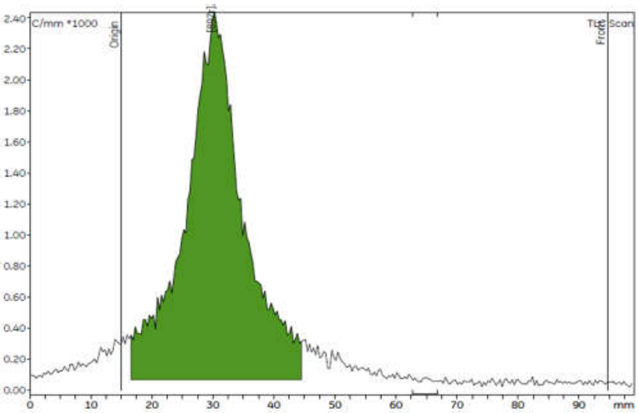


**Integration TLC Scan**

| Substance | R/F | %Total % | Type | Area Counts | %Area % | Cap fac | S/N ratio |
|---|---|---|---|---|---|---|---|
| [89Zr]Zr-oxalate | 0.196 | 82.59 | DD(M) | 25947.00 | 100.00 | 1.04 | 29.4 |
| Sum in ROI | - | - | - | 25947.00 | 100.00 | - | - |
| Total area | - | - | - | 31416.83 | - | - | - |
| Area (total) RF | - | - | - | 27680.00 | - | - | - |
| Remainder (Tot) | - | - | - | 5469.83 | 17.41 | - | - |

**(C)**

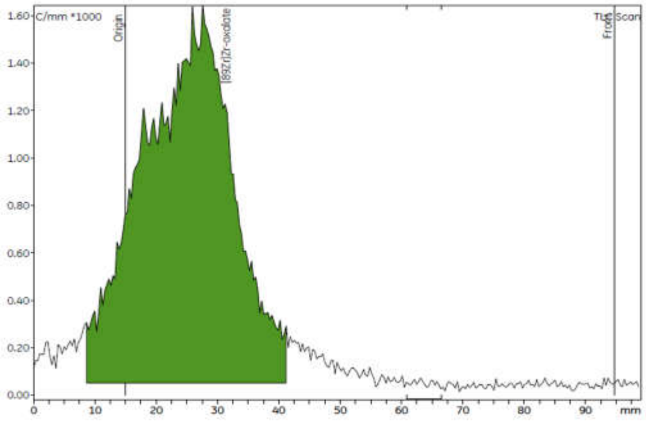


Integration TLC Scan

| Substance | R/F | %Total % | Type | Area Counts | %Area % | Cap fac | S/N ratio |
|---|---|---|---|---|---|---|---|
| [89Zr]Zr-oxalate | 0.163 | 86.44 | DD(M) | 26979.71 | 100.00 | 0.87 | 13.4 |
| Sum in ROI | - | - | - | 26979.71 | 100.00 | - | - |
| Total area | - | - | - | 31211.29 | - | - | - |
| Area (total) RF | - | - | - | 25663.65 | - | - | - |
| Remainder (Tot) | - | - | - | 4231.59 | 13.56 | - | - |

**Figure S3.** Radio-TLC chromatograms for the [$^{89}$Zr]Zr-oxalate (5.0 pH) solution using the three mobile phases: (A) 0.1M sodium citrate (method 1), (B) 20 mM citric acid (method 2), (C) methanol: ammonium acetate 1M= 7:3 (method 3)

**(A)**

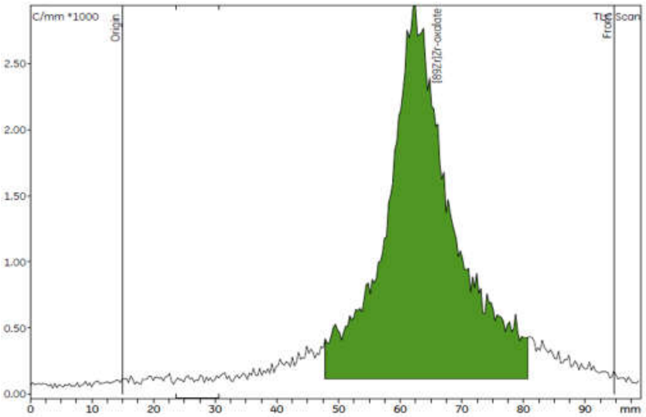


Integration TLC Scan

| Substance | R/F | %Total % | Type | Area Counts | %Area % | Cap fac | S/N ratio |
|---|---|---|---|---|---|---|---|
| [89Zr]Zr-oxalate | 0.588 | 75.30 | DD(M) | 34454.86 | 100.00 | 3.13 | 27.4 |
| Sum in ROI | - | - | - | 34454.86 | 100.00 | - | - |
| Total area | - | - | - | 45758.43 | - | - | - |
| Area (total) RF | - | - | - | 38056.71 | - | - | - |
| Remainder (Tot) | - | - | - | 11303.57 | 24.70 | - | - |

**(B)**

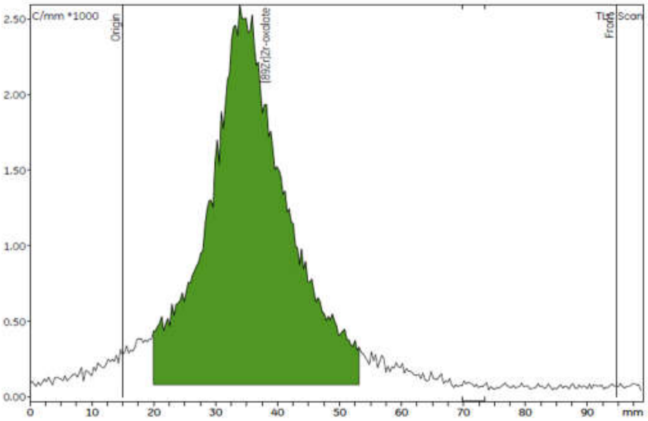


Integration TLC Scan

| Substance | R/F | %Total % | Type | Area Counts | %Area % | Cap fac | S/N ratio |
|---|---|---|---|---|---|---|---|
| [89Zr]Zr-oxalate | 0.246 | 85.83 | DD(M) | 35794.45 | 100.00 | 1.31 | 24.8 |
| Sum in ROI | - | - | - | 35794.45 | 100.00 | - | - |
| Total area | - | - | - | 41705.73 | - | - | - |
| Area (total) RF | - | - | - | 38352.09 | - | - | - |
| Remainder (Tot) | - | - | - | 5911.27 | 14.17 | - | - |

(C)

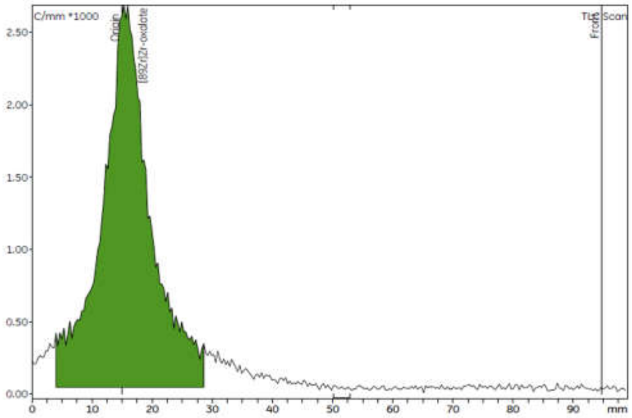


Integration TLC Scan

| Substance | R/F | %Total % | Type | Area Counts | %Area % | Cap fac | S/N ratio |
|---|---|---|---|---|---|---|---|
| [89Zr]Zr-oxalate | 0.004 | 83.28 | DD(M) | 24706.25 | 100.00 | 0.02 | 32.3 |
| Sum in ROI | - | - | - | 24706.25 | 100.00 | - | - |
| Total area | - | - | - | 29667.25 | - | - | - |
| Area (total) RF | - | - | - | 16598.00 | - | - | - |
| Remainder (Tot) | - | - | - | 4961.00 | 16.72 | - | - |

**Figure S4.** Radio-TLC chromatograms for the [$^{89}$Zr]Zr-oxalate (7.0 pH) solution using the three mobile phases: (A) 0.1M sodium citrate (method 1), (B) 20 mM citric acid (method 2), (C) methanol: ammonium acetate 1M= 7:3 (method 3)

(A)

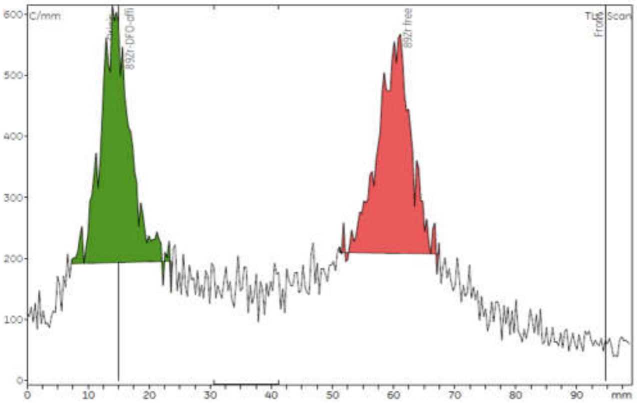


Integration TLC Scan

| Substance | R/F | %Total % | Type | Area Counts | %Area % | Cap fac | S/N ratio |
|---|---|---|---|---|---|---|---|
| 89Zr-DFO-affi | -0.008 | 16.32 | BB(M) | 2296.000 | 50.99 | -0.04 | 7.4 |
| 89Zr free | 0.575 | 15.69 | BB(M) | 2206.500 | 49.01 | 3.07 | 6.0 |
| Sum in ROI | - | - | - | 4502.500 | 100.00 | - | - |
| Total area | - | - | - | 14067.344 | - | - | - |
| Area (total) RF | - | - | - | 3373.501 | - | - | - |
| Remainder (Tot) | - | - | - | 9564.84 | 67.99 | - | - |

(B)

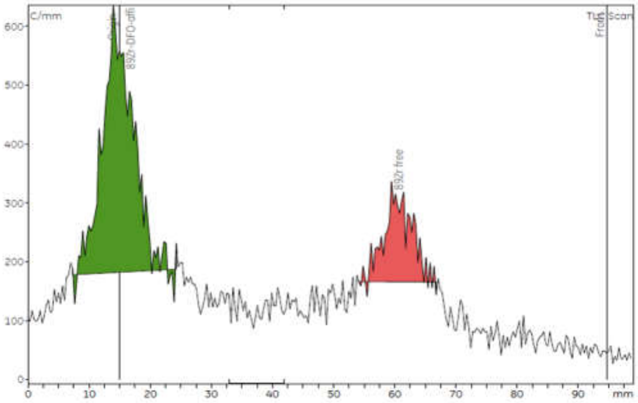


Integration TLC Scan

| Substance | R/F | %Total % | Type | Area Counts | %Area % | Cap fac | S/N ratio |
|---|---|---|---|---|---|---|---|
| 89Zr-DFO-affi | -0.004 | 22.23 | BB(M) | 2501.286 | 75.55 | -0.02 | 6.8 |
| 89Zr free | 0.567 | 7.19 | BB(M) | 809.429 | 24.45 | 3.02 | 3.0 |
| Sum in ROI | - | - | - | 3310.714 | 100.00 | - | - |
| Total area | - | - | - | 11254.036 | - | - | - |
| Area (total) RF | - | - | - | 2268.222 | - | - | - |
| Remainder (Tot) | - | - | - | 7943.32 | 70.58 | - | - |

**Figure S5.** Radio TLC chromatograms obtained after (A) 1 hour and (B) 12 hours, respectively, following the analysis of the [$^{89}$Zr]Zr-p-SCN-Bz-DFO-anti-HER2 affibody solution labelled at 7.5-8.0 pH (Experiment 3)

**(A)**

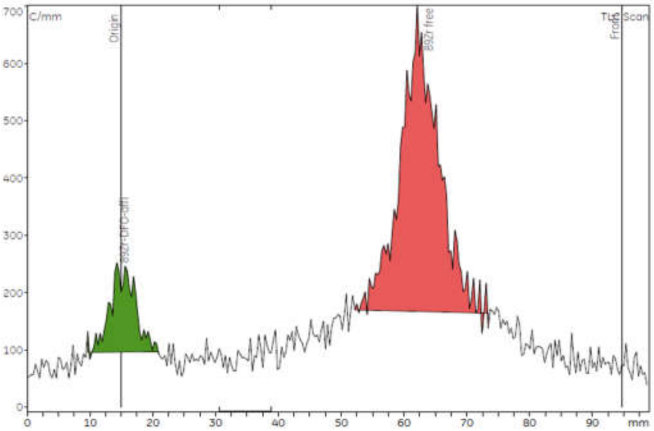


Integration TLC Scan

| Substance | R/F | %Total % | Type | Area Counts | %Area % | Cap fac | S/N ratio |
|---|---|---|---|---|---|---|---|
| 89Zr-DFO-affi | 0.000 | 5.73 | BB(M) | 705.571 | 16.35 | 0.00 | 3.1 |
| 89Zr free | 0.592 | 29.31 | BB(M) | 3609.857 | 83.65 | 3.16 | 9.9 |
| Sum in ROI | - | - | - | 4315.428 | 100.00 | - | - |
| Total area | - | - | - | 12316.760 | - | - | - |
| Area (total) RF | - | - | - | 6506.600 | - | - | - |
| Remainder (Tot) | - | - | - | 8001.33 | 64.96 | - | - |

**(B)**

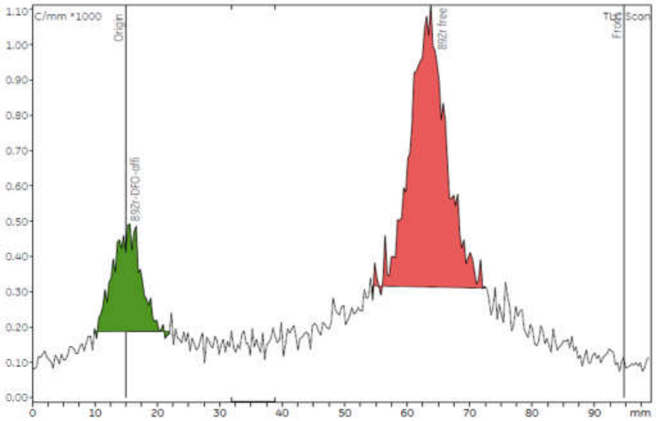


Integration TLC Scan

| Substance | R/F | %Total % | Type | Area Counts | %Area % | Cap fac | S/N ratio |
|---|---|---|---|---|---|---|---|
| 89Zr-DFO-affi | 0.013 | 7.45 | BB(M) | 1535.000 | 22.92 | 0.07 | 3.2 |
| 89Zr free | 0.608 | 25.07 | BB(M) | 5162.000 | 77.08 | 3.24 | 10.8 |
| Sum in ROI | - | - | - | 6697.000 | 100.00 | - | - |
| Total area | - | - | - | 20592.904 | - | - | - |
| Area (total) RF | - | - | - | 10360.142 | - | - | - |
| Remainder (Tot) | - | - | - | 13895.90 | 67.48 | - | - |

**Figure S6.** Radio TLC chromatograms obtained after (A) 1 hour and (B) 12 hours, respectively, following the analysis of the [$^{89}$Zr]Zr-p-SCN-Bz-DFO-anti-HER2 affibody solution labelled at 8.0-8.5 pH (Experiment 3)

**(A)**

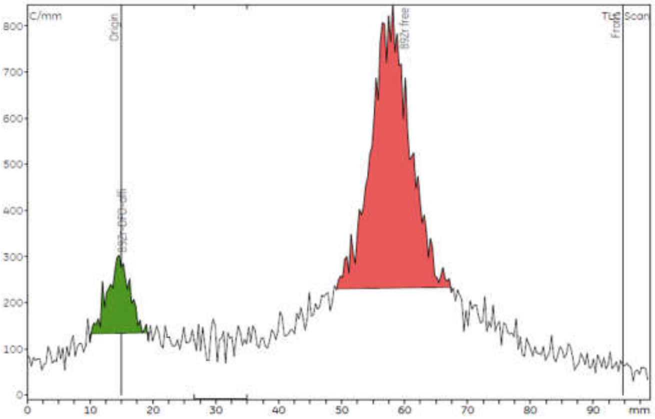


Integration TLC Scan

| Substance | R/F | %Total % | Type | Area Counts | %Area % | Cap fac | S/N ratio |
|---|---|---|---|---|---|---|---|
| 89Zr-DFO-affi | -0.004 | 4.33 | BB(M) | 663.000 | 13.41 | -0.02 | 3.3 |
| 89Zr free | 0.542 | 27.94 | BB(M) | 4279.286 | 86.59 | 2.89 | 9.9 |
| Sum in ROI | - | - | - | 4942.285 | 100.00 | - | - |
| Total area | - | - | - | 15314.560 | - | - | - |
| Area (total) RF | - | - | - | 7325.201 | - | - | - |
| Remainder (Tot) | - | - | - | 10372.27 | 67.73 | - | - |

**(B)**

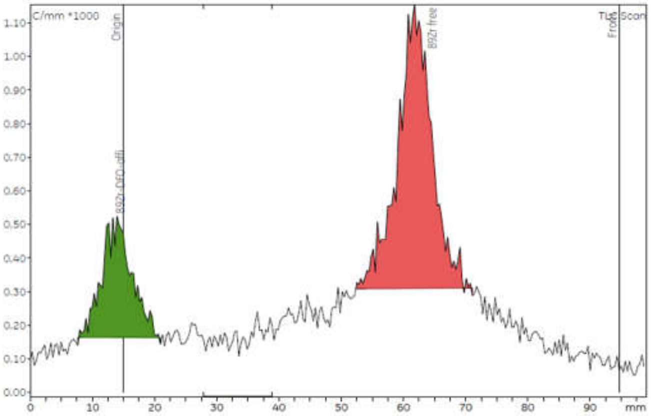


**Integration TLC Scan**

| Substance | R/F | %Total % | Type | Area Counts | %Area % | Cap fac | S/N ratio |
|---|---|---|---|---|---|---|---|
| 89Zr-DFO-affi | -0.013 | 9.31 | BB(M) | 1982.857 | 26.97 | -0.07 | 5.5 |
| 89Zr free | 0.592 | 25.22 | BB(M) | 5369.000 | 73.03 | 3.16 | 12.5 |
| Sum in ROI | - | - | - | 7351.857 | 100.00 | - | - |
| Total area | - | - | - | 21289.575 | - | - | - |
| Area (total) RF | - | - | - | 9933.181 | - | - | - |
| Remainder (Tot) | - | - | - | 13937.72 | 65.47 | - | - |

**Figure S7.** Radio TLC chromatograms obtained after (A) 1 hour and (B) 12 hours, respectively, following the analysis of the [$^{89}$Zr]Zr-p-SCN-Bz-DFO-anti-HER2 affibody solution labelled at 9.0 pH (Experiment 3)
